# Evaluation of organic load-related efficacy changes in antiseptic solutions used in hospitals

**DOI:** 10.55730/1300-0144.5379

**Published:** 2022-02-27

**Authors:** Aslı ŞAHİNER, Ece HALAT, Evren ALGIN YAPAR, Bilge Ahsen KARA

**Affiliations:** 1 Department of Biology, Faculty of Science, Ege University, İzmir, Turkey; 2 Department of Pharmaceutical Technology, Faculty of Pharmacy, Sivas Cumhuriyet University, Sivas, Turkey; 3 Ministry of Health, Ankara Gazi Mustafa Kemal Occupational and Environmental Diseases Hospital, Ankara, Turkey

**Keywords:** Antiseptic solutions, organic load in hospitals, antimicrobial efficacy, EN 13727, EN 13624

## Abstract

**Background/aim:**

In this study, it was aimed to evaluate the change in antimicrobial efficacy related to the presence of the organic load of four different antiseptic solutions that are frequently used in hospitals.

**Materials and methods:**

Solutions of hydrogen peroxide, povidone-iodine, chlorhexidine digluconate, and ethyl alcohol were prepared, tested in terms of antimicrobial efficacy changes in the presence of organic substances, and evaluated according to EN 13727 and EN 13624 standards.

**Results:**

Among the investigated solutions ethanol 70% solution showed the best results by providing a 5-log reduction on all test organisms without affecting by the type and concentration of organic substances. Solutions of hydrogen peroxide 3%, povidone-iodine 7.5%, and chlorhexidine digluconate 0.2% performed lower antimicrobial efficacy depending on the concentration of organic load.

**Conclusion:**

It is concluded that the antimicrobial efficacy of antiseptic solutions is significantly affected by the organic substances and thus the proper use of antiseptics has become important to achieve successful disinfection and prevention of antibacterial resistance.

## 1. Introduction

Antiseptic solutions are commonly used in hospitals and other healthcare settings for a variety of topical applications that kill or inhibit the growth of disease-causing bacteria. Such antiseptic solutions are biocidal products containing one or more active ingredients and are commercially produced for different purposes of use such as hand rub, hand wash, surgical solutions, mouthwashes, and also for brushes. They are expected to have properties like broad-spectrum, fast-acting, nonirritating, nontoxic, and insignificant absorption into the application site [1,2,3]. They play an important role in the prevention of nosocomial infections, which are important risk factors for morbidity and mortality especially due to antibiotic-resistant organisms in the healthcare fields [1,4,5]. It has been determined that 20%–40% of these infections are transmitted through the hands of healthcare staff touching the patient or by contamination from the environment [6]. *Staphylococcus aureus*, Methicillin-resistant *S. aureus* (MRSA), *Enterococcus faecalis, E. faecium*, Vancomycin-resistant Enterococci (VRE), broad-spectrum β-lactamase (ESBL)-producing Enterobacteriaceae, *Escherichia coli, Pseudomonas aeruginosa, Acinetobacter* spp. are the leading microorganisms associated with hospital-acquired infections [5,7,8]. *Candida albicans*, the most important fungus in terms of nosocomial infections, causes septicaemia, urinary tract infections, or surgical site infections. Antiseptic solutions containing hydrogen peroxide, chlorhexidine, triclosan, ethyl alcohol, and povidone-iodine active substances are formulated as ready-to-use or concentrated solutions for hand hygiene and wound antisepsis in the hospitals. Most antiseptics do not have a specific form of action against microorganisms as much as antibiotics, but their microbial activities show significant differences depending on many factors such as the presence of organic matter, microbial load, and synergistic effects between active substances, time, and temperature. The effects of some active substances may be reduced due to various effects, especially in the presence of organic substances, and cannot show the expected microbial reduction [2,9,10]. In addition to being used in the hospital environment, antiseptic solutions are widely used all over the world due to the Covid 19 pandemic. In this direction, it has become more important to investigate the factors affecting the effectiveness of antiseptic solutions. The failure to achieve the desired success in the use of antiseptic solutions is due to the fact that they are not used properly and under appropriate conditions. Therefore, in this study, it was aimed to examine the effect of organic load, which is a conditional factor, on the effectiveness of four antiseptic solutions that are widely used in the hospital environment. The change in antimicrobial effectiveness of prepared solutions of hydrogen peroxide (HP), povidone-iodine (PVP-I), chlorhexidine digluconate (CHX), and ethyl alcohol (EtOH), which are used as hand and wound antiseptics in hospitals were investigated in the presence of organic load at different concentrations by using phase 2 step 1 specific suspension assays and evaluated by EN 13727 [11] and EN 13624 [12] standards. There are some limitations in the applications related to the phase 2 step 2 trials on human hands; the use of only nonpathogenic *E. coli* K12 strains in artificial contamination or the inability to standardize the microbial load in studies to be constructed with natural contamination. Thus, phase 2 step 1 trials are preferred in microbial activity studies. In our study, assessment of the effectiveness of each antiseptic solution was accomplished by using four bacterial strains and yeast. The test strains included methicillin-resistant *Staphylococcus aureus* (MRSA), vancomycin-resistant *Enterococcus faecalis* (VRE), *Escherichia coli, Pseudomonas aeruginosa*, and *Candida albicans*.

## 2. Materials and methods

### Antiseptic solutions

The following antiseptic solutions were prepared and tested:

- Hydrogen peroxide (HP) (3%) was prepared from 30% hydrogen peroxide solution (CAS No.7722-84-1 0 Sigma-Aldrich) diluted with distilled water.
- Povidone-iodine (PVP-I) (7.5%) was prepared from poly(vinylpyrrolidone) (PVP)–iodine complex (CAS No. 25655-41-8 Sigma-Aldrich) diluted with citric acid-phosphate buffer solution (pH 5.0).
- Chlorhexidine digluconate (CHX) (0.2%) was prepared from a 20% stock solution (CAS No. 18472-51-0 Sigma-Aldrich) diluted with distilled water.
- Ethyl alcohol (EtOH) (70%) was prepared from 96% ethyl alcohol (CAS No. 64-17-5, Merck Millipore) diluted with distilled water.

### Test organisms

The test organisms were methicillin-resistant *Staphylococcus aureus* (ATCC 43300), vancomycin-resistant *Enterococcus faecalis* (ATCC 51299), *Pseudomonas aeruginosa* (ATCC 15442), *Escherichia coli* K12 (NCTC 10538), *Candida albicans* (ATCC 10231).

### Interfering substances

Two different organic challenges were investigated; bovine serum albumin (BSA, CAS No: 9048-46-8, Sigma Aldrich): 0.1%, 0.3%, 0.5%, 1.0%, 1.5%, 3.0%, 5.0%, 10.0%, and defibrinated sheep blood (DSB, Thermo Fisher Scientific): 0.1%, 0.3%, 0.5%, 1.0%, 1.5%, 3.0%, 5.0%, 10.0%.

### Neutralizers

Appropriate neutralizers (for EtOH: 3 g/L Lecithin, 30 g/L Saponin, 30 g/L Polysorbate 80; for HP: 10 g/L Lecithin, 0.25 g/L Catalase, 50 g/L Polysorbate 80; for PVP-I: 3 g/L Lecithin, 15 g/L Sodium Thiosulphate, 30 g/L Polysorbate 80; for CHX 3 g/L Lecithin, 30 g/L Saponin, 1 g/L L-histidine, 30 g/L Polysorbate 80) were used to inactivate the active substance residues at the end of the contact period. Neutralizer compositions are shown in Table 1. It was also tested whether neutralizers showed toxicity on microorganisms.

### 2.1. Quantitative suspension test procedure

Bactericidal and yeasticidal activity tests were performed according to EN 13727 and EN 13624 protocols, respectively [4,5]. Reference organisms were prepared by densitometer within the range of 1.5 × 10^8^ to 5.0 × 10^8^ CFU mL^−1^ for bacteria and 1.5 × 10^7^–5.0 × 10^7^ CFU mL^−1^ for yeast for 18–24 h.

Bovine serum albumin (BSA) and the defibrinated sheep blood (DSB) were prepared at the above concentrations just prior to testing. One mL of interfering substances were transferred into sterile tubes and 1 mL of each culture suspension were added and waited for 2 min. After the 2-minute equilibration, 8 mL of antiseptic solutions (×1.25 of the final test concentration) were added and waited for the 1 min of contact time. At the end of contact time, aliquots of 1 mL were transferred to appropriate neutralization solutions (Table 1). After 5 min of neutralization time, 0.5 mL of test mixtures and serial dilutions (10^−1^ and 10^−2^) were plated on appropriate agar medium (TSA for bacteria, MEA for yeast) in duplicates. After incubation for 48 h (37 °C for bacteria and 30 °C for fungi) colonies were counted. In addition, interfering substance and neutralization controls (test validations) were applied as described in EN standards.

### 2.2. Calculation of reduction

The logarithmic reduction was calculated according to EN standards using the following formula;


$$\text{lgR}={\text{lgN}}_{0}-{\text{lgN}}_{\text{a}}$$

lg N_0_ = number of colonies at the beginning of contact time
lg Na = number of colonies at the end of contact time

## 3. Results

The results showed that depending on the organic load investigated, four different antiseptic solutions presented different effectiveness. Antimicrobial activities of antiseptic solutions were tested on 4 different bacteria and 1 yeast, including antibiotic-resistant strains, at different concentrations of 2 different organic substances BSA and DSB. One-min period specified in the EN standards was applied as contact time. Bactericidal and yeasticidal efficacy data obtained in the tests are below presented in separate tables for each test solution (Table 1–4). According to EN 13624 and 13727 standards, bactericidal and yeasticidal efficacy limits are 5 log and 4 log, respectively. When the effectiveness of antiseptic solutions was compared under standard test conditions, the efficacy of active ingredients other than ethyl alcohol was reduced in the presence of gradually increasing organic load.

**Figure 1:**
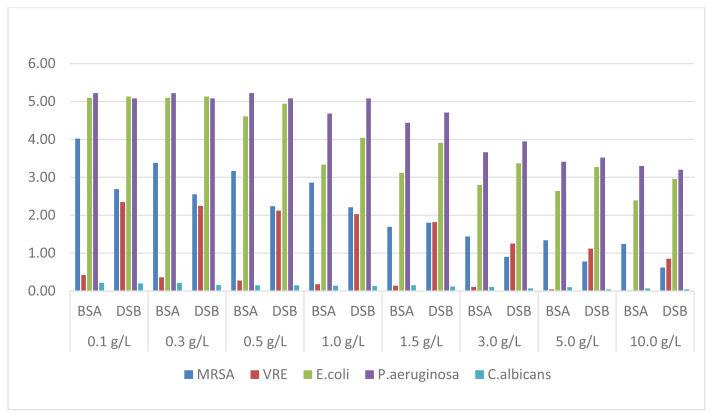
Reduction factors obtained with HP-3% solution.

HP 3% solution showed 5-log reduction only on *P. aeruginosa* and *E. coli*. However, this effect gradually decreased when the BSA concentration increased above 0.5% and the DSB concentration above 1%. A limit value for MRSA, VRE, and *C.albicans* was not achieved in any trial and almost no effect was observed especially in the presence of high organic substances (Figure 1).

**Figure 2:**
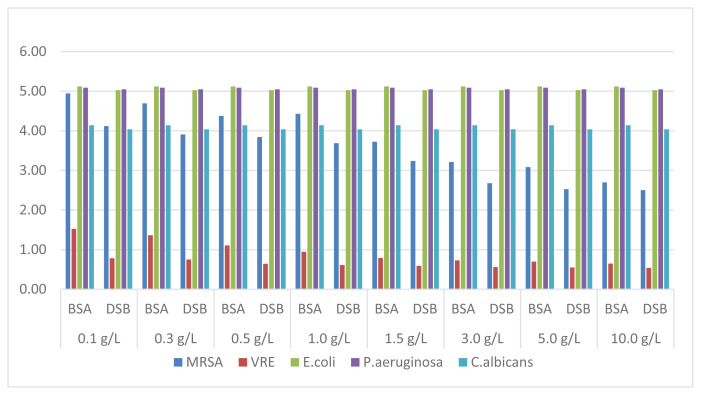
Reduction factors obtained with PVP-7.5% solution.

7.5% PVP-I solution provided the desired 5-log and 4-log reduction in standards against gram negative bacteria and *C. albicans*, respectively, similar to HP 3% solution, but the efficacy did not reach the desired value for MRSA and especially VRE (Figure 2).

**Figure 3:**
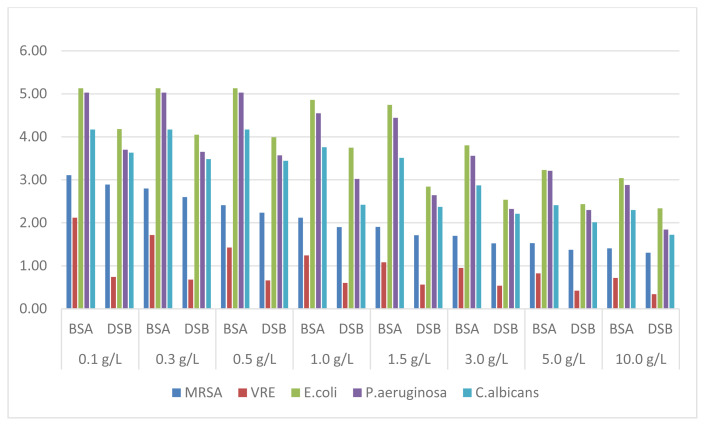
Reduction factors obtained with CHX-0.2% solution.

CHX-0.2% solution, which provided a 5-log reduction in *P. aeruginosa* and *E. coli* and a 4-log reduction in *C.albicans* up to 0.5% BSA concentration, showed a lower efficacy in the presence of DSB. In the presence of a high organic load, an activity varying between 1.72 and 3.04 log was achieved against these organisms. While this antiseptic provided a maximum reduction of around 3 log in MRSA, the reduction in VRE was determined to be at most 2 log (Figure 3).

**Figure 4:**
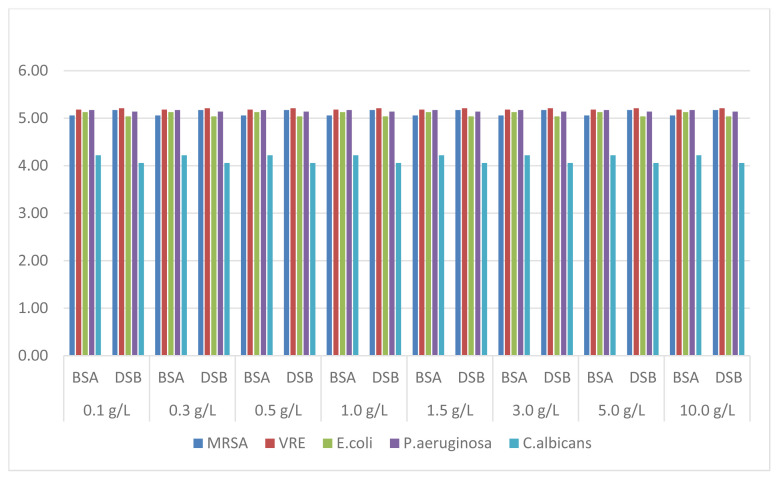
Reduction factors obtained with EtOH-70% solution.

In our efficacy trials, EtOH-70% was observed to be the antiseptic solution that provided the logarithmic reduction to meet the standards in all test organisms and at each concentration value (Figure 4).

## 4. Discussion

When the results were evaluated, the highest efficacy was found with the ethanol including EtOH-70% solution. The factors determining the degree of effectiveness in other active substances were both the organic load and the type of microorganism. The response of antiseptic solutions to organic substances varies in relation to the chemical structures of their active ingredients. The action mechanism of antiseptic solutions plays a key role in varying effectiveness against different microorganisms [13]. It has been observed that especially antibiotic-resistant strains are more resistant to antiseptic solutions. To determine the bactericidal and fungicidal activities of antiseptic solutions, BSA and DSB are used interfering substances to simulate organic contamination in the wound surface or skin tissue, according to EN methods. A test model was designed to determine the response of different antiseptic solutions in the presence of 2 different types of organic load and their varying concentrations to make more accurate predictions about the potential performance of an antiseptic solution in clinical use.

Obtained results showed that HP 3% solution provided the desired 5-log reduction in the standard only at low concentrations of organic substances in *P. aeruginosa* and *E. coli* bacteria, and the efficiency decreased inversely as the amount of organic substance increased. While a 4-log reduction was achieved on MRSA in the presence of 0.1 g/L BSA, this effect decreased to around 1 log with increasing concentrations of BSA. In the presence of DSB, the effectiveness was observed to be less. It was determined that HP did not show any antimicrobial activity against VRE and *C. albicans*, especially in the presence of BSA (Figure 1), which is in accordance with the results of the study in which the antimicrobial activity against *E. faecium* and *C. albicans* was investigated [9]. HP is a biocidal active agent with a wide range of uses, from antisepsis to disinfection and equipment sterilization, due to its environmental friendliness and rapid conversion to harmless products such as oxygen and water. HP degrades microbial structure by producing hydroxyl free radicals (−OH) that attack key cell components, including proteins, enzymes, lipids, and DNA. However, its biggest disadvantage is that it is rapidly reduced in the presence of organic substances and decomposed by radical scavenger enzymes such as catalase and peroxidase, thus it does not show any effect at low concentrations and in short periods against microorganisms possess these enzymes.

Aqueous or alcoholic (tincture) solutions of iodine have been used as antiseptics for over 150 years. By the development of iodine carrier or release iodophors, some disadvantages such as irritability and instability of iodine have been overcome [14,15]. PVP-I, in which elemental iodine forms a complex with the polyvinylpyrrolidone carrier, has a rapid effect against bacteria, yeasts, viruses, and protozoa, as the effect is very low against molds and spores [2]. PVP-I is frequently used in the medical field as a disinfectant or topical antiseptic in the form of solution, powder, or lotion formulations. High antimicrobial activity against bacteria and yeasts and relatively lower efficacy against *A. brasiliensis* of PVP-I solution have been reported [3]. Although its mechanism of action has not been fully elucidated, it is thought to cause deterioration of the function and structure of the cell by reacting with the functional groups of amino acids, nucleotides, and fatty acids in the cell membrane, cell wall, and cytoplasm [1,14]. Enveloped viruses are more sensitive to iodophors than other viruses. Similar to bacteria, iodine attacks the surface proteins of enveloped viruses, as well as destabilizes the membrane by reacting with the carbon bonds in the unsaturated fatty acids of the membrane [15]. Our results showed that 7.5% PVP-I solution reduced 5 logs of *E. coli* and *P. aeruginosa* and 4 logs of *C. albicans* under the investigated conditions related to organic substances. While a 4-log reduction was achieved in low BSA and blood concentrations against MRSA, it was determined that this effect regressed to around 2.5 log as the concentration increased. VRE was noted as the most resistant organism to PVP-I solution. While only 1.52-log reduction was obtained at the lowest BSA concentration, this efficacy decreased inversely with the BSA concentration. When DFB is used as the interfering substance, even 1-log reduction in the organism could not be achieved (Figure 2).

In the case of CHX solution, the results showed that it was the antiseptic most significantly affected by organic substance type and its concentration. When DFB was used as the organic substance, a relatively lower effect was observed compared to BSA. Antimicrobial activity decreased significantly with increasing concentration. Similar to the results with other antiseptic solutions, the most resistant strain to CHX was found to be VRE (Figure 3). CHX is a broad-spectrum biocide used both as a hand sanitizer and an oral antiseptic, formed by chlorine binding to two guanidine. In addition to its advantages such as being nonirritant and long-lasting effect on the skin, its effectiveness varies highly depending on pH and the concentration of organic load. CHX salts are positively charged and therefore tightly bound to the negatively charged bacterial cell wall and membrane. This binding results in the death of the cell as it causes deterioration in the bacterial wall and membrane structure. Some researchers have also found that high concentrations of CHX inhibit the ATPase enzyme [14,16].

Alcohols, especially ethanol (at 60%–80% concentration), are active ingredients that are frequently used in the medical field as both antiseptic and disinfectant. The antimicrobial mechanism of ethanol is to disrupt membrane integrity and denature proteins. The hydrogen bonding of the hydroxyl group (−OH) in alcohols to proteins results in the loss of structure and function of microbial proteins and enzymes. Although alcohols have a broad-spectrum effect, they are not sporicidal but have sporostatic activity [14,17]. According to the results obtained by ethanol 70% solution, it provided a 5-log reduction on all test organisms. It has been determined that ethanol 70% solution is not affected by the type of organic substances and its concentration used, and the efficiency continues without decreasing even at the highest organic substance concentration (Figure 4). In the experiments, globular proteins albumin and haemoglobin were used as interfering substances. The organic solvent ethanol denatures proteins at high concentrations because of hydrophobic interactions. Therefore, it is thought that ethanol may not be affected by interfering agents [18].

In our study, changes in the microbiological activities of the most commonly used antiseptic solutions in the hospitals were observed against different organic interfering substances and their different concentrations. It has been determined that among the active substances used as antiseptics, other than ethyl alcohol, there are changes in their effectiveness at different levels depending on the concentration of organic load, and there is a decrease in their antimicrobial activities inversely proportional to the increasing concentration organic substances. It has been observed that MRSA and VRE, which play an important role in nosocomial infections and cannot be treated easily due to their antibiotic resistance, are more resistant to antiseptics than other tested microorganism strains, and that other active substances other than ethanol, especially against VRE, cannot provide successful efficacy even at low organic load concentrations. On the other hand, Pitten et al. [9] determined that the microbial efficiency of antiseptics containing oxidizing agents is higher when there is no organic load. In addition to organic load, it is known that the contact time is an important variable on the effectiveness, the effectiveness increases in direct proportion to the contact time, but this time is limited to 5 min in the EN 13727 standard for antiseptic products used in the medical field on the human body [3].

Improper use of antiseptics or disinfectants causes microorganisms to not be eliminated, activates resistance genes, increases bacterial tolerance, and leads to phenotypic adaptations. In addition, resistance genes can be horizontally transferred and cause other organisms to become resistant. The growth rate of disinfectant-resistant bacteria is very high, which reduces the effectiveness of disinfectants. Multidrug-resistant bacteria ultimately pose a serious threat to human and environmental health [19,20].

## 5. Conclusion

The use of antiseptic and disinfectant solutions in healthcare settings, ensuring the hygiene of patients and healthcare staff, play a key role in the prevention of nosocomial infections and bacterial resistance. To get optimum efficiency from antiseptic and disinfectant solutions, attention should be paid to the active ingredients, usage area, and contact time. The effectiveness of many antiseptic products decreases depending on the organic load in the environment. Improper use of antiseptics resulted in a decrease in their efficacy and increased bacterial tolerance. Organic load-related changes in the activity of antiseptics could be controlled by deciding proper antiseptics and ethyl alcohol-included antiseptics could overcome high organic load risk related to unwanted bacterial resistance.

## Figures and Tables

**Table 1:** Reduction factors obtained with HP-3% solution.

Interfering Substance		MRSA	VRE	*E. coli*	*P. aeruginosa*	*C. albicans*
**0.1 g/L**	BSA	4.02 ± 0.13	0.42 ± 0.04	5.10 ± 0.11	5.22 ± 0.10	0.22 ± 0.17
	DSB	2.69 ± 0.22	2.35 ± 0.18	5.13 ± 0.13	5.08 ± 0.08	0.20 ± 0.12
**0.3 g/L**	BSA	3.38 ± 0.04	0.36 ± 0.12	5.10 ± 0.21	5.22 ± 0.02	0.21 ± 0.16
	DSB	2.55 ± 0.11	2.25 ± 0.24	5.13 ± 0.15	5.08 ± 0.05	0.16 ± 0.10
**0.5 g/L**	BSA	3.17 ± 0.30	0.28 ± 0.25	4.61 ± 0.11	5.22 ± 0.03	0.15 ± 0.13
	DSB	2.24 ± 0.12	2.12 ± 0.24	4.94 ± 0.24	5.08 ± 0.05	0.15 ± 0.15
**1.0 g/L**	BSA	2.86 ± 0.16	0.18 ± 0.26	3.34 ± 0.13	4.68 ± 0.20	0.14 ± 0.62
	DSB	2.21 ± 0.08	2.03 ± 0.07	4.04 ± 0.16	5.08 ± 0.06	0.13 ± 0.45
**1.5 g/L**	BSA	1.69 ± 0.10	0.14 ± 0.11	3.12 ± 0.06	4.44 ± 0.07	0.15 ± 0.33
	DSB	1.80 ± 0.20	1.82 ± 0.33	3.91 ± 0.22	4.71 ± 0.24	0.12 ± 0.30
**3.0 g/L**	BSA	1.44 ± 0.16	0.11 ± 0.35	2.80 ± 0.24	3.66 ± 0.14	0.11 ± 0.38
	DSB	0.90 ± 0.25	1.25 ± 0.10	3.37 ± 0.18	3.95 ± 0.16	0.07 ± 0.30
**5.0 g/L**	BSA	1.34 ± 0.32	0.04 ± 0.20	2.64 ± 0.25	3.41 ± 0.22	0.10 ± 0.23
	DSB	0.78 ± 0.22	1.12 ± 0.24	3.27 ± 0.30	3.52 ± 0.25	0.04 ± 0.26
**10.0 g/L**	BSA	1.24 ± 0.11	0.02 ± 0.24	2.39 ± 0.04	3.30 ± 0.07	0.07 ± 0.34
	DSB	0.62 ± 0.30	0.85 ± 0.23	2.96 ± 0.18	3.20 ± 0.10	0.05 ± 0.25

**Table 2:** Reduction factors obtained with PVP-7.5% solution.

Interfering Substance		MRSA	VRE	*E. coli*	*P. aeruginosa*	*C. albicans*
**0.1 g/L**	BSA	4.94 ± 0.22	1.52 ± 0.10	5.12 ± 0.05	5.09 ± 0.06	4.14 ± 0.12
	DSB	4.12 ± 0.20	0.78 ± 0.32	5.03 ± 0.02	5.05 ± 0.04	4.04 ± 0.04
**0.3 g/L**	BSA	4.69 ± 0.14	1.37 ± 0.42	5.12 ± 0.07	5.09 ± 0.08	4.14 ± 0.07
	DSB	3.91 ± 0.22	0.75 ± 0.24	5.03 ± 0.22	5.05 ± 0.03	4.04 ± 0.05
**0.5 g/L**	BSA	4.38 ± 0.15	1.11 ± 0.21	5.12 ± 0.06	5.09 ± 0.07	4.14 ± 0.10
	DSB	3.84 ± 0.24	0.64 ± 0.26	5.03 ± 0.12	5.05 ± 0.02	4.04 ± 0.13
**1.0 g/L**	BSA	4.43 ± 0.26	0.95 ± 0.25	5.12 ± 0.04	5.09 ± 0.07	4.14 ± 0.12
	DSB	3.69 ± 0.28	0.61 ± 0.36	5.03 ± 0.02	5.05 ± 0.12	4.04 ± 0.11
**1.5 g/L**	BSA	3.73 ± 0.32	0.79 ± 0.34	5.12 ± 0.07	5.09 ± 0.06	4.14 ± 0.05
	DSB	3.24 ± 0.24	0.59 ± 0.27	5.03 ± 0.04	5.05 ± 0.05	4.04 ± 0.04
**3.0 g/L**	BSA	3.22 ± 0.18	0.73 ± 0.25	5.12 ± 0.05	5.09 ± 0.11	4.14 ± 0.07
	DSB	2.68 ± 0.26	0.56 ± 0.28	5.03 ± 0.02	5.05 ± 0.06	4.04 ± 0.08
**5.0 g/L**	BSA	3.09 ± 0.12	0.70 ± 0.38	5.12 ± 0.11	5.09 ± 0.07	4.14 ± 0.06
	DSB	2.53 ± 0.16	0.55 ± 0.42	5.03 ± 0.08	5.05 ± 0.06	4.04 ± 0.12
**10.0 g/L**	BSA	2.70 ± 0.20	0.65 ± 0.35	5.12 ± 0.12	5.09 ± 0.06	4.14 ± 0.10
	DSB	2.50 ± 0.24	0.54 ± 0.22	5.03 ± 0.10	5.05 ± 0.07	4.04 ± 0.02

**Table 3:** Reduction factors obtained with CHX-0.2% solution.

Interfering Substance		MRSA	VRE	*E. coli*	*P. aeruginosa*	*C. albicans*
**0.1 g/L**	BSA	3.11 ± 0.36	2.12 ± 0.22	5.13 ± 0.06	5.03 ± 0.05	4.17 ± 0.10
	DSB	2.89 ± 0.18	0.74 ± 0.31	4.18 ± 0.26	3.70 ± 0.36	3.63 ± 0.05
**0.3 g/L**	BSA	2.80 ± 0.26	1.72 ± 0.21	5.13 ± 0.04	5.03 ± 0.11	4.17 ± 0.07
	DSB	2.60 ± 0.18	0.68 ± 0.24	4.05 ± 0.20	3.65 ± 0.12	3.48 ± 0.08
**0.5 g/L**	BSA	2.41 ± 0.32	1.43 ± 0.14	5.13 ± 0.05	5.03 ± 0.10	4.17 ± 0.19
	DSB	2.24 ± 0.17	0.66 ± 0.35	3.99 ± 0.20	3.57 ± 0.27	3.44 ± 0.11
**1.0 g/L**	BSA	2.12 ± 0.29	1.24 ± 0.30	4.86 ± 0.27	4.55 ± 0.24	3.76 ± 0.25
	DSB	1.90 ± 0.23	0.60 ± 0.34	3.75 ± 0.30	3.02 ± 0.17	2.42 ± 0.14
**1.5 g/L**	BSA	1.91 ± 0.27	1.08 ± 0.35	4.74 ± 0.17	4.44 ± 0.05	3.51 ± 0.13
	DSB	1.71 ± 0.24	0.56 ± 0.22	2.84 ± 0.21	2.64 ± 0.18	2.37 ± 0.16
**3.0 g/L**	BSA	1.70 ± 0.21	0.95 ± 0.24	3.80 ± 0.15	3.56 ± 0.20	2.87 ± 0.25
	DSB	1.52 ± 0.22	0.54 ± 0.24	2.54 ± 0.23	2.32 ± 0.38	2.21 ± 0.34
**5.0 g/L**	BSA	1.53 ± 0.20	0.83 ± 0.30	3.23 ± 0.15	3.21 ± 0.13	2.41 ± 0.25
	DSB	1.37 ± 0.30	0.42 ± 0.16	2.44 ± 0.20	2.30 ± 0.10	2.01 ± 0.30
**10.0 g/L**	BSA	1.40 ± 0.25	0.72 ± 0.14	3.04 ± 0.12	2.88 ± 0.22	2.30 ± 0.16
	DSB	1.30 ± 0.31	0.34 ± 0.38	2.34 ± 0.21	1.84 ± 0.24	1.72 ± 0.38

**Table 4:** Reduction factors obtained with EtOH-70% solution.

Interfering Substance		MRSA	VRE	*E. coli*	*P. aeruginosa*	*C. albicans*
**0.1 g/L**	BSA	5.06 ± 0.06	5.18 ± 0.07	5.13 ± 0.13	5.17 ± 0.15	4.22 ± 0.07
	DSB	5.17 ± 0.04	5.21 ± 0.12	5.04 ± 0.16	5.14 ± 0.11	4.06 ± 0.15
**0.3 g/L**	BSA	5.06 ± 0.10	5.18 ± 0.05	5.13 ± 0.04	5.17 ± 0.12	4.22 ± 0.11
	DSB	5.17 ± 0.13	5.21 ± 0.16	5.04 ± 0.06	5.14 ± 0.10	4.06 ± 0.07
**0.5 g/L**	BSA	5.06 ± 0.07	5.18 ± 0.07	5.13 ± 0.14	5.17 ± 0.12	4.22 ± 0.05
	DSB	5.17 ± 0.11	5.21 ± 0.04	5.04 ± 0.05	5.14 ± 0.06	4.06 ± 0.10
**1.0 g/L**	BSA	5.06 ± 0.06	5.18 ± 0.11	5.13 ± 0.14	5.17 ± 0.04	4.22 ± 0.05
	DSB	5.17 ± 0.12	5.21 ± 0.11	5.04 ± 0.16	5.14 ± 0.04	4.06 ± 0.07
**1.5 g/L**	BSA	5.06 ± 0.10	5.18 ± 0.12	5.13 ± 0.07	5.17 ± 0.07	4.22 ± 0.08
	DSB	5.17 ± 0.04	5.21 ± 0.14	5.04 ± 0.06	5.14 ± 0.05	4.06 ± 0.05
**3.0 g/L**	BSA	5.06 ± 0.05	5.18 ± 0.10	5.13 ± 0.14	5.17 ± 0.16	4.22 ± 0.10
	DSB	5.17 ± 0.06	5.21 ± 0.14	5.04 ± 0.11	5.14 ± 0.12	4.06 ± 0.06
**5.0 g/L**	BSA	5.06 ± 0.10	5.18 ± 0.05	5.13 ± 0.00	5.17 ± 0.02	4.22 ± 0.11
	DSB	5.17 ± 0.06	5.21 ± 0.06	5.04 ± 0.07	5.14 ± 0.07	4.06 ± 0.12
**10.0 g/L**	BSA	5.06 ± 0.07	5.18 ± 0.12	5.13 ± 0.10	5.17 ± 0.06	4.22 ± 0.14
	DSB	5.17 ± 0.11	5.21 ± 0.16	5.04 ± 0.05	5.14 ± 0.04	4.06 ± 0.05
